# Spawning aggregation of white-streaked grouper *Epinephelus ongus*: spatial distribution and annual variation in the fish density within a spawning ground

**DOI:** 10.7717/peerj.3000

**Published:** 2017-02-14

**Authors:** Atsushi Nanami, Taku Sato, Yuuki Kawabata, Junichi Okuyama

**Affiliations:** 1 Research Center for Sub-tropical Fisheries, Seikai National Fisheries Research Institute, Japan Fisheries Research and Education Agency, Ishigaki, Okinawa, Japan; 2 Research Center for Marine Invertebrates, National Research Institute of Fisheries and Environment of Inland Sea, Japan Fisheries Research and Education Agency, Onomichi, Hiroshima, Japan; 3 Graduate School of Fisheries and Environmental Sciences, Nagasaki University, Nagasaki, Nagasaki, Japan

**Keywords:** Spawning aggregation, Grouper, Coral reef fish, *Epinephelus ongus*

## Abstract

White-streaked grouper (*Epinephelus ongus*) is an important fisheries target and forms spawning aggregations at particular spawning grounds. The aims of the present study were to investigate the ecological characteristics of annual spawning aggregations such as (1) spatial variations in the density of *E. ongus* at the spawning ground, (2) the relationship between fish density and environmental variables, (3) inter-annual variations in the spawning aggregation, (4) the proportion of males to females at the spawning ground for several days pre—and post-spawning and (5) the relationship between male density and female density at the protected spawning ground, based on observations over five years at an Okinawan coral reef. Although the protected spawning ground area was large (ca. 2,500 m × 700 m), high density of *E. ongus* (over 25 individuals per 100 m^2^) was found in a limited area (within c.a. 750 m × 50 m). Current velocity and coverage of rocks had significant positive effects on the spatial distribution of *E. ongus* at the spawning ground. Inter-annual variation in the degree of aggregation was found and this variation was explained by the annual variation of mean seawater temperature during 40 days before the spawning day. The male–female ratio (male:female) at the spawning ground was ca. 3:1 for three years (May 2012, May 2014 and May 2015) whereas >13:1 for one year (May 2013). Significant positive relationships between male density and female density were found at the aggregation sites. It is suggested that *E. ongus* use aggregation sites with greater current velocity to reduce the risk of egg predation and seawater temperature is one of the main factors that is responsible for determining the degree of aggregation. It is also suggested that females possibly select sites with a greater density of males and this selection behavior might be the reason why females arrived at the spawning ground after the arrival of the males. For effective management of spawning grounds, precise site selection as well as the duration of the protection period are suggested to be key aspects to protect the spawning aggregations of *E. ongus*, which have been currently achieved at the spawning ground.

## Introduction

Coral reef fishes are highly diverse and at least 80 species have been reported to form spawning aggregations among over 2,600 fish species (Sadovy de Mitcheson & Colin, 2012). Domeier (2012) has defined that reef fish spawning aggregations consist of only conspecific individuals and that spawning is highly predictable in time and space. Spawning aggregations are usually found in restricted seasons and lunar phases at particular sites (Nemeth, 2009). Spawning aggregations can be categorized into two types such as resident spawning aggregations and transient spawning aggregations (Domeier & Colin, 1997). Resident spawning aggregations are characterized by a short migration distance (<2 km), small size species (e.g., Acanthuridae, Caesionidae, Labridae and Scaridae), short duration of the spawning event (1–5 h) and high spawning frequency (daily or monthly). In contrast, transient spawning aggregations are characterized by a long migration distance (2 to >100 km), large size species (e.g., Lethrinidae, Epinephelidae and Lutjanidae), long duration of the spawning event (>2 to 10 days) and low spawning frequency (annually) (Nemeth, 2009).

Among the transient spawning aggregation species, over 40 species of groupers (Epinephelidae) are regarded to form spawning aggregations (Sadovy de Mitcheson & Colin, 2012). Some ecological characteristics of groupers in terms of spawning aggregation have been studied such as migration distance (Nemeth et al., 2007; Nanami, Ohta & Sato, 2015), spawning migration behavior (Zeller, 1998; Zeller & Russ, 1998; Starr et al., 2007; Rhodes et al., 2012), spawning behavior (Colin, 1992; Samoilys & Squire, 1994; Donaldson, 1995; Sadovy, 1996; Nanami et al., 2013a), reproductive activity (Robinson et al., 2008; Ohta & Ebisawa, 2015) and location of spawning aggregations (Kobara & Heyman, 2010; Golbuu & Friedlander, 2011; Colin, 2012; Erisman et al., 2012). For fisheries aspects, the effective protection of the spawning aggregations of groupers is needed (Beets & Friedlander, 1999; Sala, Ballesteros & Starr, 2001; Nemeth, 2005; Sadovy & Domeier, 2005; Russell, Luckhurst & Lindeman, 2012) due to their predictability in time and space and vulnerability to fishing (Samoilys, 1997; Rhodes & Tupper, 2008; Sadovy de Mitcheson et al., 2008; Sadovy de Mitcheson & Erisman, 2012).

**Figure 1:**
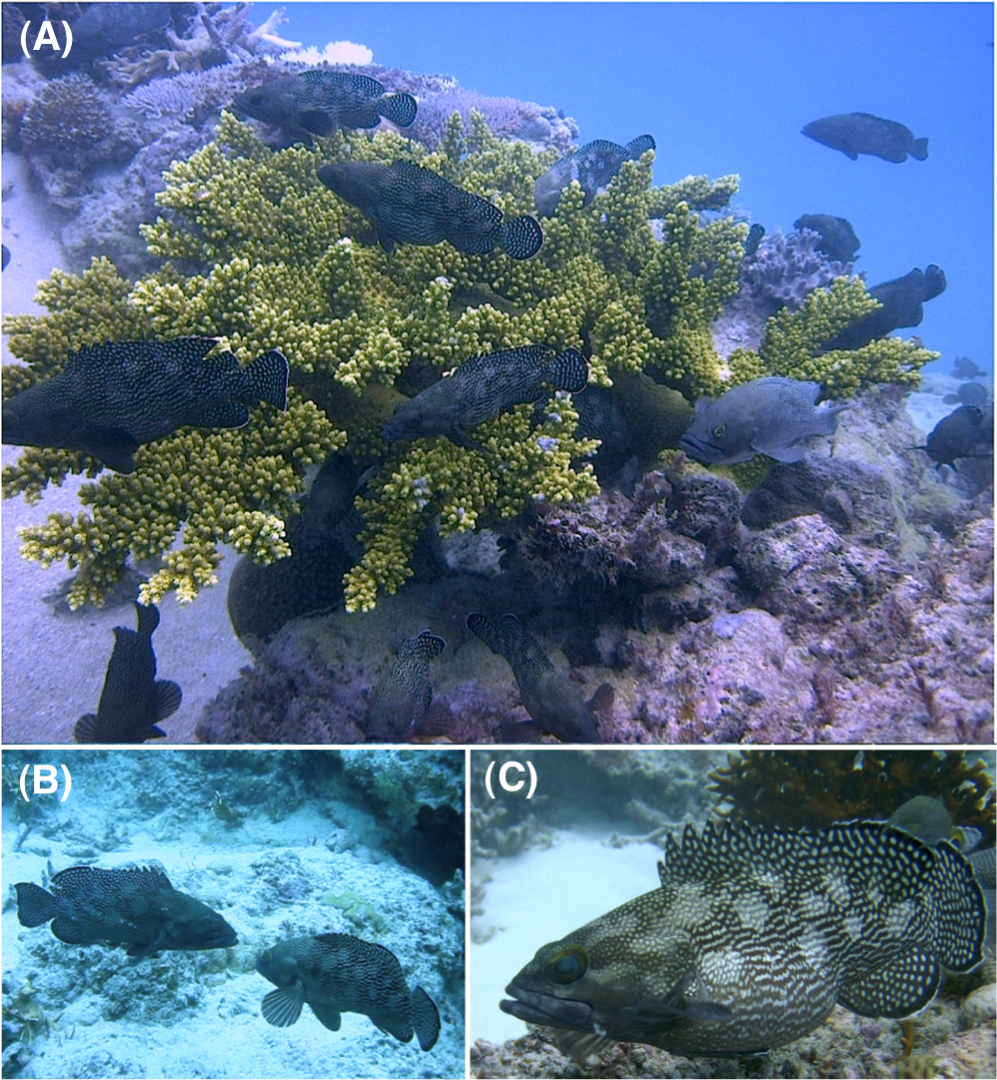
Underwater photographs of the spawning aggregation of *Epinephelus ongus* at the Yonara Channel (spawning ground), showing aggregation (A), agonistic display between two males (B) and a female with expanded abdomen due to developed ovaries (C). Photographs were taken at the last-quarter moon phase in May 2012 (A) and May 2013 (B, C).

White-streaked grouper *Epinephelus ongus* is one of the important fisheries targets around the Okinawan region (Ohta & Ebisawa, 2016) and known to form spawning aggregations in the region (Ohta & Ebisawa, 2009; Ohta & Ebisawa, 2015; Ohta & Nanami, 2009; Nanami et al., 2013a; Nanami et al., 2014; Nanami, Ohta & Sato, 2015) (Fig. 1, Videos S1 and S2). Ohta & Ebisawa (2009); Ohta & Ebisawa (2015) examined the reproductive biology using gonadal histology, oocyte development and catch data analysis. They showed clear seasonality of spawning of *E. ongus* and the spawning was found during the last-quarter moon in only one month (only May) or two consecutive months (April–May or May–June). Spawning migration behavior (Nanami et al., 2014; Kawabata et al., 2015) and spawning migration distance (Nanami, Ohta & Sato, 2015) have also been clarified for *E. ongus*. However, other ecological aspects such as spatial variation in the density within the spawning ground in relation to environmental characteristics, annual variations in the degree of aggregation, the proportion of males to females at the spawning ground and male–female relationship (e.g., relationship between male density and female density at spawning ground) remain unknown. Understanding the precise ecological characteristics of spawning aggregations would be useful for effective management of *E. ongus* including the necessary scale and duration of spawning ground protection.

The aims of the present study were to investigate the ecological characteristics of the spawning aggregations of *E. ongus.* Specifically, the aims were to clarify (1) spatial variations in the density of *E. ongus* at the spawning ground, (2) the relationship between fish density and environmental variables, (3) inter-annual variations in the spawning aggregation, (4) the proportion of males to females at the spawning ground around the spawning days and (5) the relationship between male density and female density at the protected spawning ground, based on observations at an Okinawan coral reef.

**Figure 2:**
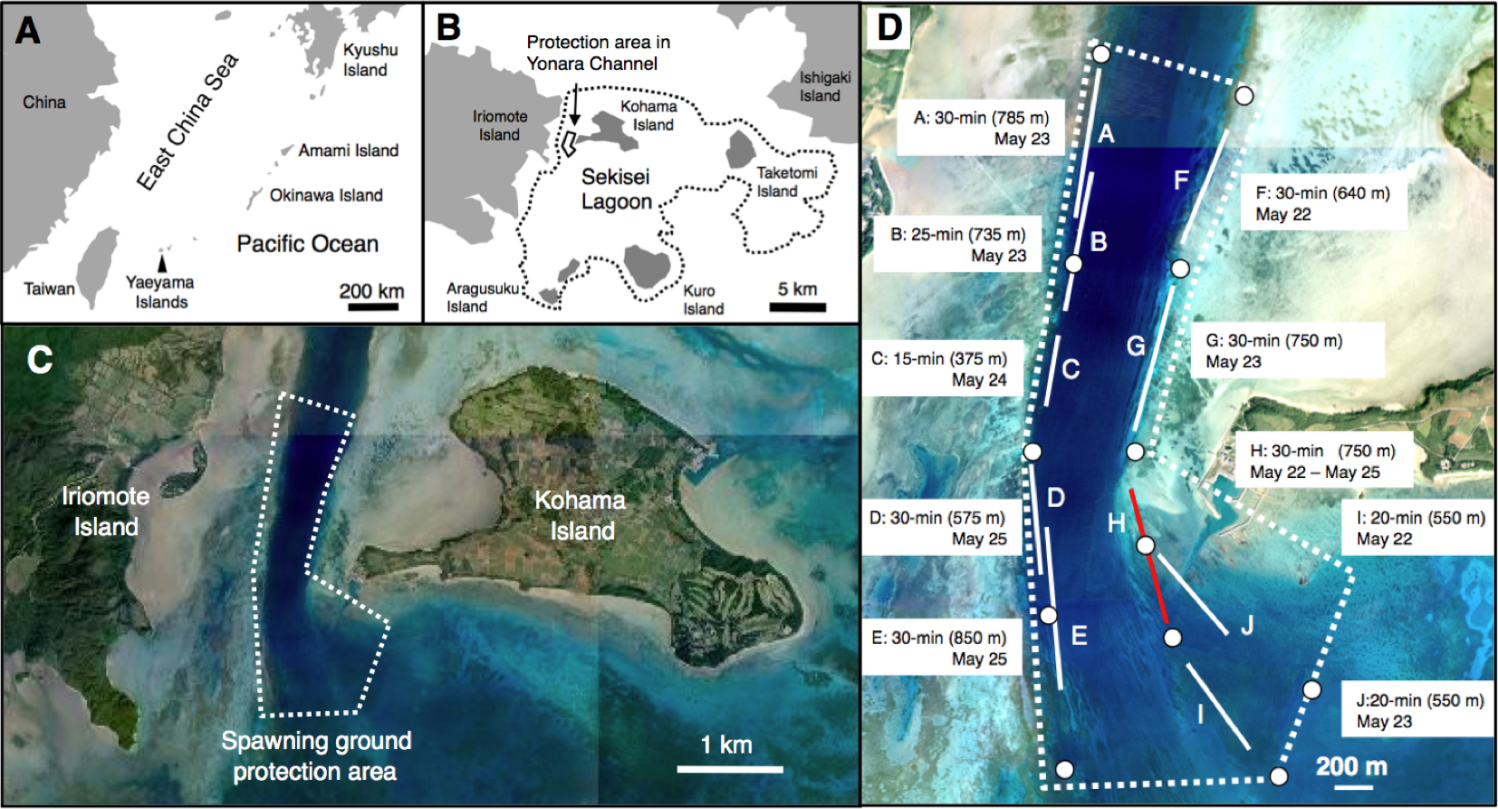
Map of the study site. Map showing the position of the Yaeyama Islands (A), Sekisei lagoon (B) (enclosed by a dotted line), the spawning ground protection area (C) (enclosed by a dotted line) and the location of ten transect (A–J) to clarify the spatial distribution of *Epinephelus ongus* in May 2011 (=LCM 5) (D). White circles in (D) represent the setting positions where the current velocities were measured using plaster balls. Since the density of *E. ongus* was highest on one transect (transect H, red line), this transect was defined as “750 m × 5 m control transect.” Aerial photographs in (C) and (D) were provided by International Coral Reef Research and Monitoring Center.

## Materials and Methods

The study was conducted using field observations of free-living fishes in their natural habitat. No sampling procedure was conducted in the present study.

### Study site and species

*Epinephelus ongus* is a protogynous hermaphrodite and has a longevity of up to 20 years (Ohta & Ebisawa, 2016) and mating occurs as paired spawners that release gametes during vertical swimming towards to surface (Nanami et al., 2013a). This study was conducted at Sekisei lagoon in the Yaeyama Islands, Okinawa, located in the southernmost part of Japan (Figs. 2A, 2B). One of the main spawning grounds of *E. ongus* is located at Yonara Channel (Ohta & Nanami, 2009; Nanami et al., 2013a; Nanami et al., 2014; Nanami, Ohta & Sato, 2015) (Figs. 2B, 2C). The Yonara Channel was confirmed as a spawning ground of *E. ongus* (Ohta & Nanami, 2009; Nanami et al., 2013a; Nanami et al., 2014). This spawning ground has been protected during spawning periods since 2010. The protected area of the spawning ground is ca. 2,500 m × 700 m (Fig. 2C).

**Figure 3:**
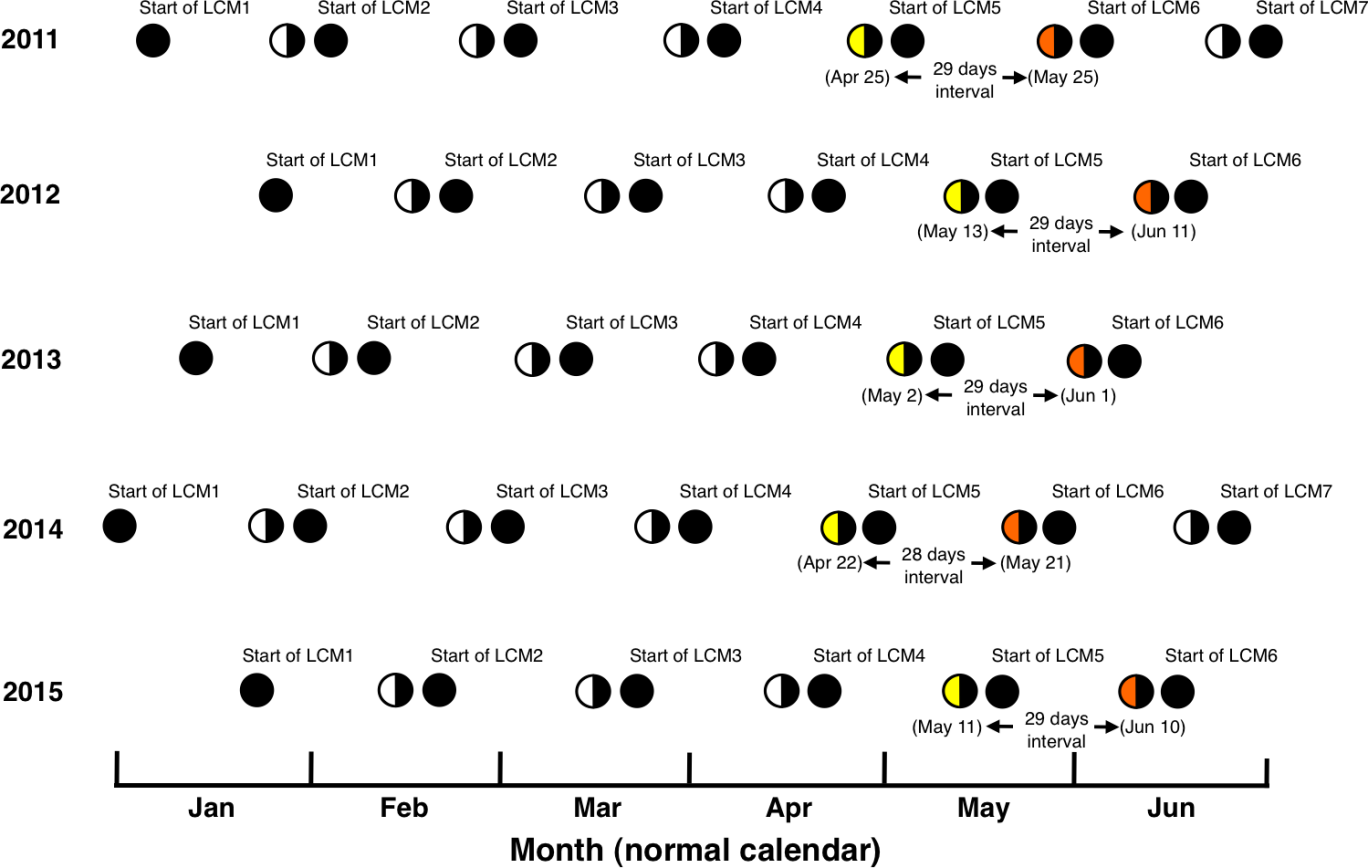
Definition of “Lunar calendar month” (LCM) and the last-quarter moon phase at LCM 4 and LCM 5 (colored by yellow and orange, respectively). The definition for the five years (2011–2015) is shown. Black circles and white semi-circles represent the new moon and last-quarter moon, respectively. The date of normal calendar (Gregorian) is also shown on the last-quarter moon phase at LCM 4 and LCM 5.

Ohta & Ebisawa (2015) have studied the reproductive biology of *E. ongus* and a clear reproductive periodicity in relation to the lunar cycle was found as follows. Ohta & Ebisawa (2015) defined the lunar cycle that the first new moon of the year was the first day of the first lunar calendar month (defined as LCM 1, Fig. 3). Each LCM consists of 28–30 days and the lunar days for the last-quarter moon was day 23 or 24. Ohta & Ebisawa (2015) found that gonadsomatic index (GSI) peaks and developed gonads were only found in the last-quarter moon phase in LCM 4 and/or LCM 5. Thus, it can be concluded that spawning occurs in the last-quarter moon phase at one (LCM 4 or LCM 5) or two consecutive lunar months (LCM 4 and LCM 5).

### Spatial distribution at the spawning ground in the spawning period

#### Fish survey

In order to clarify the spatial variation in the density at the spawning ground, daytime underwater observations (09:00 h–15:00 h) were conducted at ten transects (A–I) to cover the whole spawning ground around last quarter moon in April (LCM 4) and May (LCM 5) 2011 (Figs. 2C, 2D). Since no *E. ongus* individuals were found in April 2011 (LCM 4), the data obtained in May 2011 (LCM 5) was used for the analysis. Since it was not possible to collect data for all ten transects within one day due to logistic constraints, 4 days were taken for data collection for all ten transects (May 22–May 25). At each transect, a 15 to 30-minute underwater observation using SCUBA were conducted (Fig. 2D). The number of individuals was counted per every 1 min. The width of each transect was set as 5 m. During the observations, a portable GPS receiver, sealed in a waterproof case and attached to a buoy, was towed and the course and distance of the tracks were obtained (Colin, 2012). The mean distance was 25.0 m ± (1.1 m standard error) per 1 min. The depth range at which the underwater observations were conducted was 5–16.5 m.

For analysis, each transect was divided into 5-min sub-transects. Then, the number of individuals of *E. ongus* counted and the distance for the 5-min sub-transect were obtained from the GPS tracking data. From the data, the number of individuals was converted to density (20 m × 5 m) and plotted on the map of the spawning ground.

#### Collection of environmental variables

Fifteen environmental variables were collected (13 categories of substrates, current velocity and water depth). Data for 13 categories of substrates were recorded in accordance with Nanami et al. (2013b). In short, a waterproof digital camera was attached to the frame of data collection sheet and video images were recorded during the underwater observation of fishes. In the laboratory, for every 5-second interval, a static image was obtained using QuickTime Player software (c.a. 2 m-intervals on average). The substrate at the center of each static image was recorded from the image on the monitor of a personal computer. The substrates were classified into 13 categories: (1) branching *Acropora*, (2) branching *Millepora*, (3) tabular *Acropora*, (4) massive corals (*Porites* spp. and Faviidae spp.), (5) encrusting corals, (6) other corals (corals with extremely low coverage such as branching *Pocillopora*, branching *Porites* and bottlebrush *Acropora* were pooled), (7) soft corals, (8) dead branching *Acropora*, (9) dead branching *Millepora*, (10) dead tabular *Acropora*, (11) coral rubble (scattered dead coral fragments), (12) rocks (calcium carbonate substrates having low structural complexity) and (13) sand. For analysis, the data of substrates were pooled for the each of the above-mentioned 5-min sub-transects (i.e., 60 static images per one 5-min sub-transect) and regarded as the substrate coverage for each sub-transect.

The current velocity was measured using plaster balls in accordance with Komatsu & Kawai (1992) and Yokoyama, Inoue & Abo (2004). The plaster balls are manufactured by Doris Japan (Tokyo, Japan: http://www.doris.co.jp/plaster.html). The diameter of each plaster ball is 48 mm and an iron bolt with 250 mm length and 6 mm diameter is attached in each plaster ball. Twelve plaster balls were attached with diving weight (2 kg) and set in the spawning ground between 10:40 h and 13:39 h and they were collected between 09:25 h and 11:18 h in the next day (the 12 setting positions are shown in Fig. 2D). The height between the plaster balls and substrate was set as 20 cm. The mean current velocity during the setting time (c.a. 22 h on average: range =20.9 –23.8 h) was calculated by the decrease rate in terms of weight, setting time and seawater temperature using the formula of Yokoyama, Inoue & Abo (2004). Since the current velocity changes in accordance with the lunar phase (e.g., spring tide or neap tide) and season, all year round absolute current velocity could not be measured. Thus, current velocity obtained within the c.a. 22 h was applied for analysis as degree of spatial difference in current velocity among the sites (defined as “relative current velocity”). In order to obtain the relative current velocity for each 5-min sub-transect, interpolation was applied by distance using the setting points of the plaster balls and the numerical values of current velocity at the setting points.

The water depth was measured by a diving computer (Xtender Quattro, SCUBAPRO). The diving computer recorded the depth profile with a 30-second interval. The clocks between the diving computer and GPS receiver were synchronized with each other. The depth profiles were downloaded into a personal computer and analyzed using PCLogBook software (SCUBAPRO). Thus, a 10 point depth profile at 30-second intervals was obtained for each 5-min sub-transect (2 point depth per 1 min × 5 min). For analysis, the mean point depth for each 5-min sub-transect was used for the analysis.

#### Data analysis for spatial distribution

Stepwise multiple regression analysis was performed to clarify the relationship between density of *E. ongus* (dependent variable) and the above-mentioned 15 environmental variables (independent variables). Prior to the analysis, the substrate coverage data was arc-sin transformed. Since some environmental variables showed significant positive or negative correlation coefficients with each other among the 15 environmental variables, principal component analysis (PCA) was performed to reduce the number of independent variables for multiple regression analysis (Quinn & Keough, 2002) using PRIMER software (version 6). PCA scores for five PC axes were selected due to higher eigenvalues (>1). Then, stepwise multiple regression analysis was performed in which the PCA scores for the five PC axes are independent variables using StatView software. No significant correlation coefficients were found among the five PC axes. Prior to the analysis, the density data were log (*x* + 1) transformed. The coefficient of determination (*R*^2^) was used as a criterion for goodness-of-fit.

### Inter-annual variation in the density at the spawning ground

From the results of the spatial distribution in May 2011, it was found that density of the species was extremely high at transect H (see Results: the location of transect H is shown in Fig. 2D). In the present study, therefore, the transect H was defined as “750 m × 5 m control transect.” Inter-annual underwater observations were conducted at the 750 m × 5 m control transect during several days around the last-quarter moon in LCM 4 and LCM 5 between 2011 and 2015. The start and end of the 750 m × 5 m control transect were marked by small buoys. Additional buoys were set at c.a. 50-m intervals between the start and end points. This procedure allowed the diver (Nanami A.) to swim the same route on the 750 m ×5 m control transect for every survey. The observations were conducted by using SCUBA equipment.

In order to clarify the relationship between inter-annual variations in the density and seawater temperature, seawater temperature profiles during 40 days before the spawning aggregation were obtained (provided by International Coral Reef Research and Monitoring Center, Ministry of Environment, Japan). Then, mean seawater temperature during the 40 days before the last-quarter moon was calculated and plotted against the maximum number of *E. ongus* at the 750 m × 5 m control transect.

### Male–female ratio and relationship between male density and female density at the spawning ground

In order to clarify the daily changes in the male and female density during the spawning period, 20 m × 5 m transects were established around the 750 m × 5 m control transect. Five 20 m × 5 m transects were established in 2012 and an additional five 20 m × 5 m transects were established in 2013, 2014 and 2015 (ten 20 m × 5 m transects in total for the three years). Each 20 m × 5 m transect was fixed throughout the four years of surveys with small buoys at the start and the end of each transect. Sex of *E. ongus* could be identified by their body shape characteristics in the spawning aggregation in the spawning ground. Namely, individuals with a slender body and with an expanded abdomen were males and females, respectively (Nanami et al., 2013a). The underwater observations were started four days before the last-quarter moon and continued until no females were found on all 20 m × 5 m transects.

The male–female ratio was expressed as the proportion of males to females (male:female). In this procedure, female density was converted into 1 and daily change of the proportion was obtained. Simple linear regression analysis was conducted to obtain the relationship between male density and female density.

## Results

### Spatial distribution at the spawning ground in the spawning period

**Figure 4:**
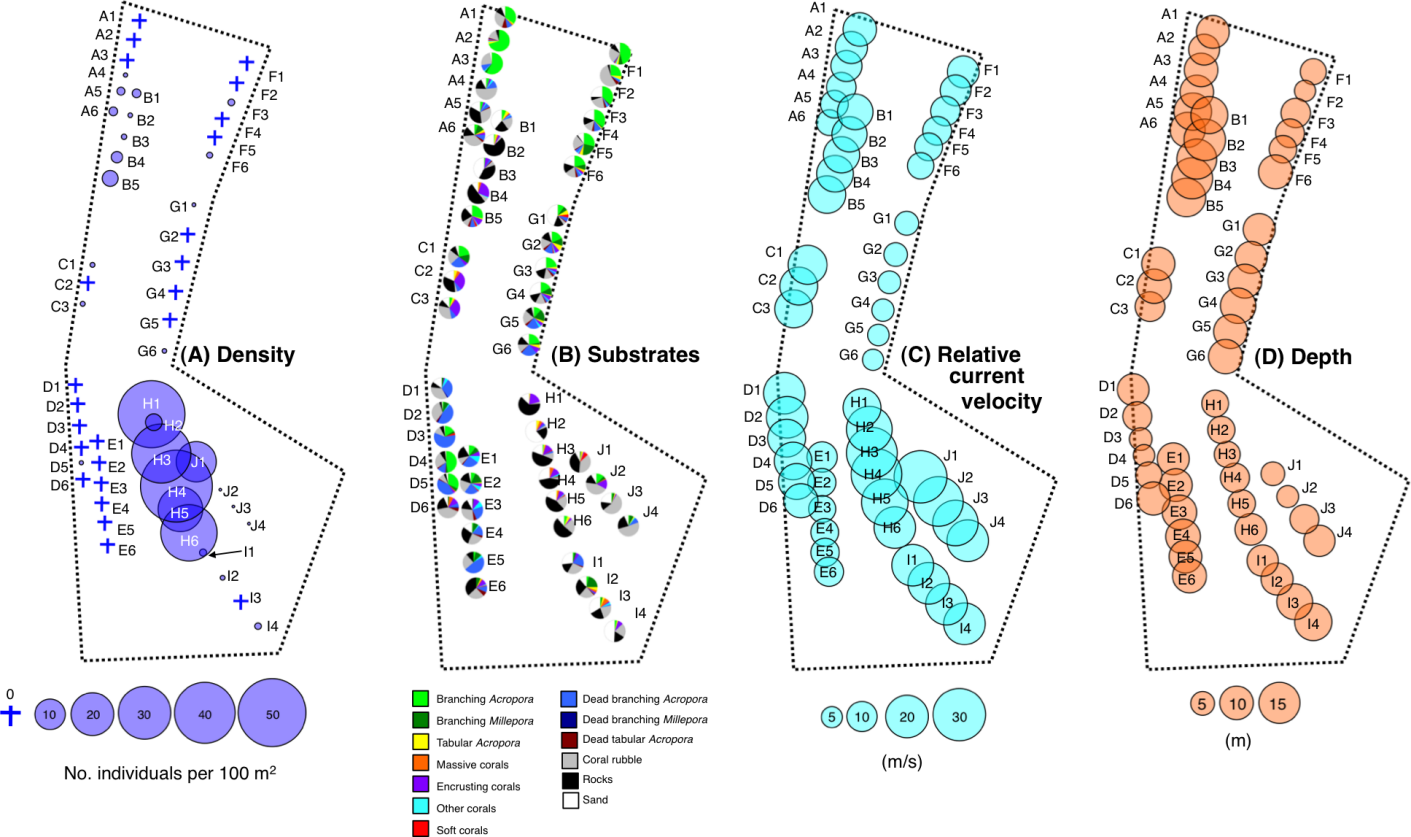
Spatial distribution of the *Epinephelus ongus* in the spawning ground in the spawning period (May 2011 = LCM 5) (A), the substrate characteristics (B), relative current velocity (C) and depth (D). Since each transect was divided into 5-minute sub-transects, they are represented as alphabet letters and numbers (for example, since transect A was 30-minute transect, it could to be divided into six 5-minute sub-transects and the six sub-transects were assigned as A1, A2, A3, A4, A5 and A6).

There was clear spatial variation in the density of *Epinephelus ongus* at the spawning ground in the spawning period (Fig. 4A). High density of *E. ongus* was found along five 5-min sub-transects on transect H (H1 and H3–H6: 22.6–58.0 individuals per 100 m^2^) and one 5-min sub-transect on transect J (J1: 29.1 individuals per 100 m^2^). These sub-transects were located at the convex-shaped edge of the Yonara channel (Fig. 2D). In contrast, 0–3.11 individuals per 100 m^2^ were found at the other eight transects (transect A–G and I). For substrate characteristics, branching *Acropora*, dead branching *Acropora*, coral rubble, rocks and sand were the major substrate components in the spawning ground (Fig. 4B). The branching *Acropora* were commonly found at the northern part (transect A and F) and southwestern part (sub-transect D4 and D5). In contrast, coral rubble, rocks and sand were the major substrate at the southeastern part (transect H, I and J). Relative current velocity was the greatest at transect H and J, intermediate at transect A, B, C, D and I and the lowest at transect E, F and G (Fig. 4C).

**Figure 5:**
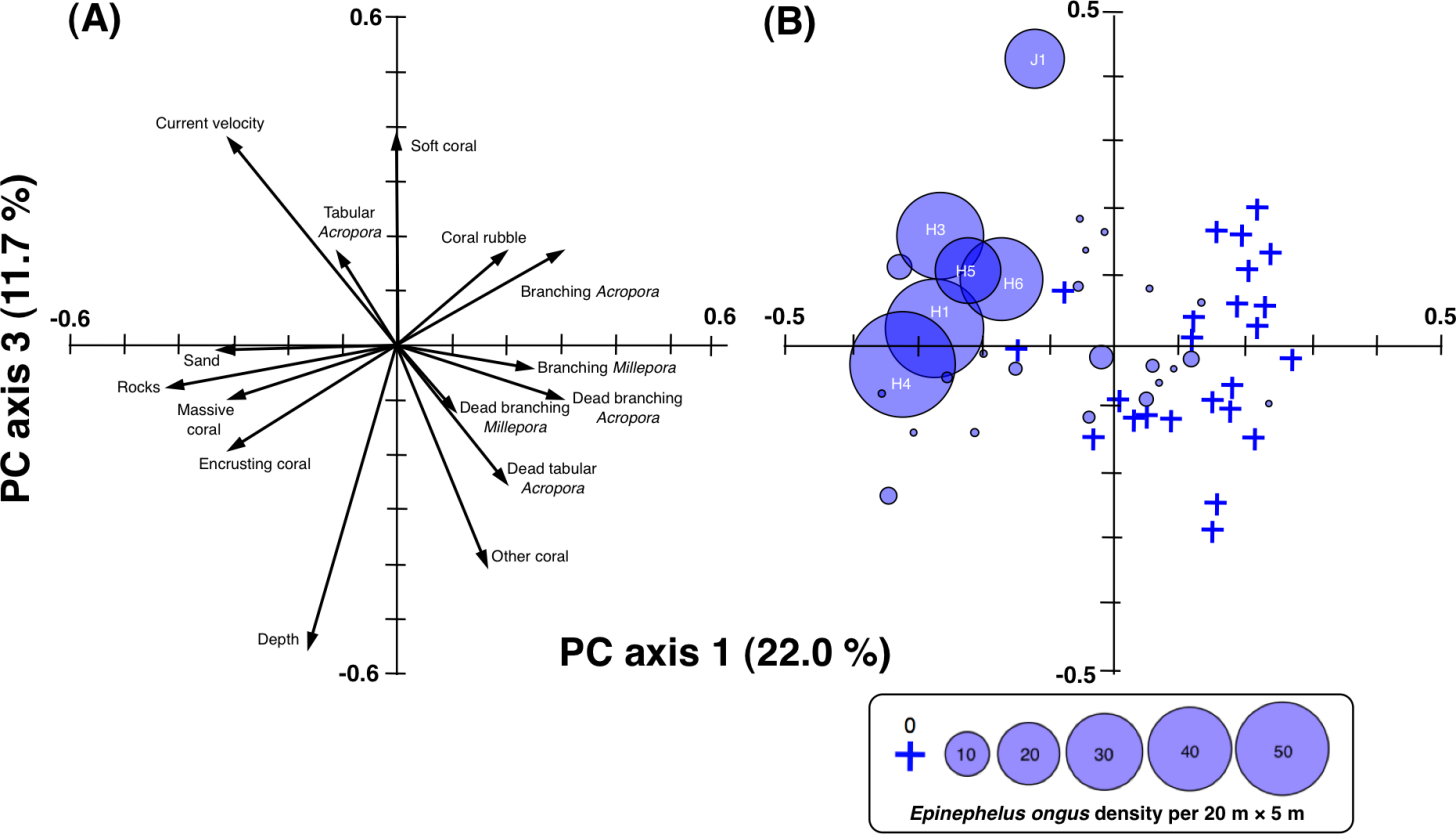
Result of principal component analysis (PCA) showing the relationship between 15 environmental variables and quadrants (A) and PCA scores of 5-minute sub-transects (B) on PC axis 1 and PC axis 3. Significant contribution for these two axes to the spatial distribution of *Epinephelus ongus* at the spawning ground was found by stepwise multiple regression analysis (Table 2). The density of *E. ongus* and the sub-transects numbers with greater density (H1, H3–H6 and J1) are simultaneously shown in (B). Since other three PC axes (PC axis 2, PC axis 4 and PC axis 5) did not have significant standard regression coefficients for the stepwise multiple regression analysis, the two-dimensional plots in terms of these non-significant three PC axes are not shown.

The results of PCA revealed the relationship between 15 environmental characteristics and five PC axes (Table 1). Multiple regression analysis revealed that PC axis 1 and PC axis 3 showed significant negative and positive standard regression coefficients, respectively (Table 2). Two-dimensional plotting of 15 environmental variables on PC axis 1 and PC axis 3 revealed that the second quadrant (i.e., upper left quadrant with negative values of PC axis 1 and positive values of PC axis 3) was mainly characterized as positive effect of relative current velocity and greater coverage of rocks (Fig. 5A). Two-dimensional plotting of PCA scores for sub-transects also revealed that the five sub-transects having greater density of *E. ongus* (H1, H3, H5, H6 and J1) were plotted at the second quadrant, indicating the positive effect of relative current velocity and coverage of rocks on the greater density of *E. ongus* (Fig. 5B, Fig. S1) and negative effect of depth (Figs. 4D, 5B, Fig. S1).

### Inter-annual variation in the density at the spawning ground

**Figure 6:**
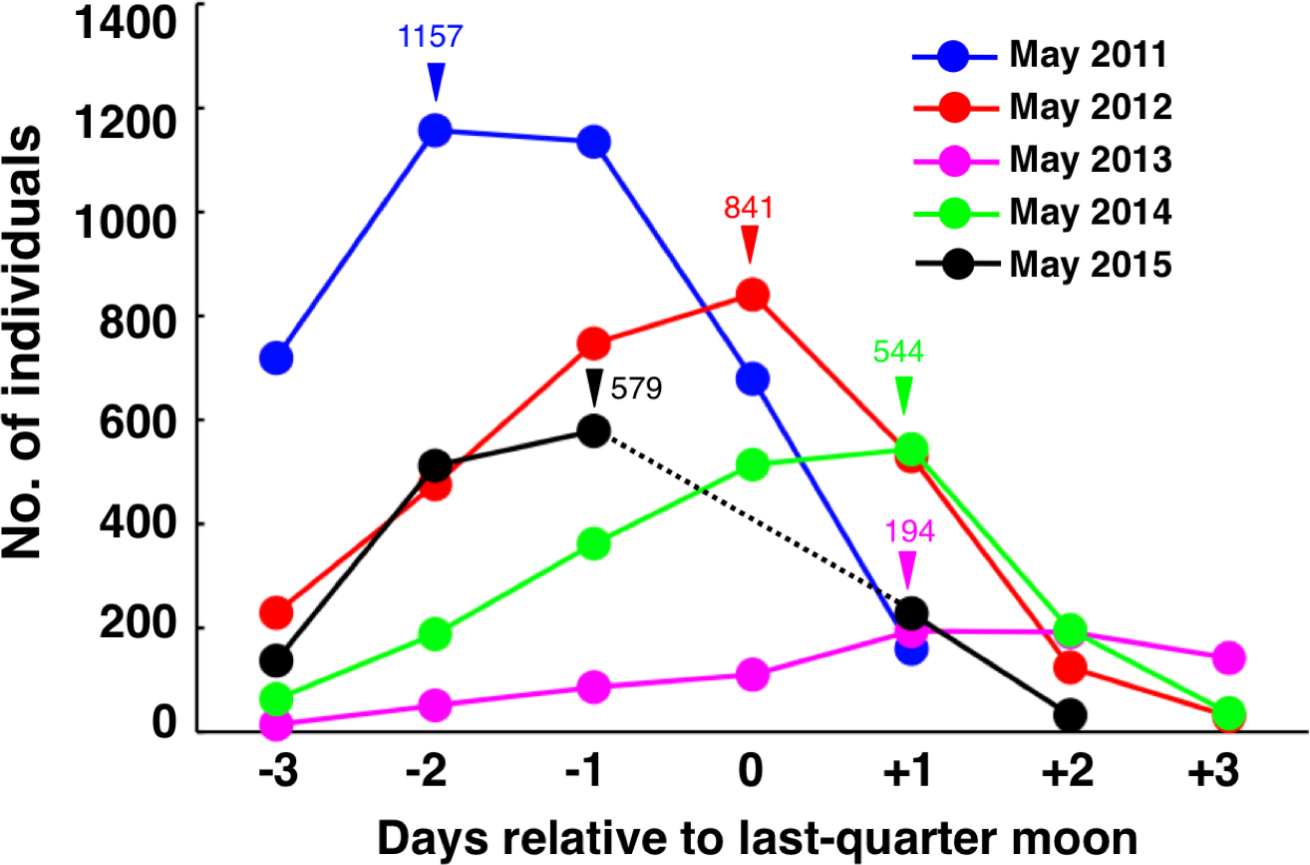
Inter-annual variation in the density of *Epinephelus ongus*. Inter-annual variation in the density of *Epinephelus ongus* on 750 m × 5 m control transect (transect H, see Fig. 2D). Inverted triangles and numbers represent the day that highest density was found and number of individuals at that time, respectively. Data could not be collected due to the typhoon that hit at the last-quarter moon in May 2015.

The highest numbers of individuals at the spawning ground were only found in May at the core site between 2011 and 2015 (Table 3). The peak density of *E. ongus* was found around the last-quarter moon at the spawning period (Fig. 6). The peak density was highest in May 2011 (30.85 individuals per 100 m^2^), and lowest in May 2013 (5.17 individuals per 100 m^2^). The number of individuals per 100 m^2^ ranged from 14.51 to 22.43 individuals in May 2012, May 2014 and May 2015 (Fig. 6).

**Table 1:** Results of principal component analysis. Results showing the 15 environmental variables and five principal component axes. Variables with the highest loadings (eigenvectors) for each PC axis are indicated in bold characters.

Environmental variables	PC axis 1	PC axis 2	PC axis 3	PC axis 4	PC axis 5
Branching *Acropora*	0.302	−0.343	0.174	−0.201	0.378
Branching *Millepora*	0.254	−0.064	−0.043	0.290	**−0.613**
Tabular *Acropora*	−0.112	**−0.452**	0.158	0.014	−0.513
Massive corals	−0.317	0.140	−0.095	−0.261	−0.255
Encrusting corals	−0.312	0.073	−0.201	0.093	0.190
Other corals	0.170	0.136	−0.399	0.295	−0.055
Soft corals	−0.004	−0.131	0.397	0.378	0.000
Dead branching *Acropora*	0.317	0.284	−0.098	0.384	0.066
Dead branching *Millepora*	0.096	0.435	−0.123	−0.208	−0.200
Dead tabular *Acropora*	0.204	−0.182	−0.252	**−0.414**	−0.040
Coral rubble	0.202	0.320	0.175	−0.396	−0.191
Rocks	**−0.427**	0.032	−0.070	0.190	0.106
Sand	−0.335	−0.167	−0.017	−0.113	−0.133
Relative current velocity	−0.302	0.387	0.382	0.016	−0.049
Depth	−0.165	−0.169	**−0.553**	0.019	−0.019
Eigenvalues	3.30	1.98	1.75	1.28	1.11
Variance (%)	22.0	13.2	11.7	8.5	7.4
Cumulative variance (%)	22.0	35.2	46.9	55.4	62.8

**Table 2:** The relationship between *Epinephelus ongus* density and five principal component axes (predictor) at the spawning ground in the spawning period (May 2011). The relationship between *Epinephelus ongus* density and five principal component axes (predictor) at the spawning ground in the spawning period (May 2011) using stepwise multiple regression analysis ( *R*^2^ = 0.439 , *p* < 0.00001 ).

Predictor	Standard regression coefficient	*p* -value
PC axis 1	−0.567	< 0.00001
PC axis 3	0.329	0.0030

**Table 3:** Number of *Epinephelus ongus* at last-quarter moon at the 750 m 5 m control transect in LCM 4 and LCM 5 (LCM: lunar calendar month) The number of *Epinephelus ongus* at last-quarter moon at the 750 m × 5 m control transect in LCM 4 and LCM 5 (LCM: lunar calendar month), which were expected to find spawning aggregation of *Epinephelus ongus* at the present study site in accordance with Ohta & Ebisawa (2015). The dates of the normal calendar (Gregorian) are also shown for the lastquarter moon phase.

Year	LCM 4		LCM 5	
2011	0	(April 25)	**679**	**(May 25)**
2012	**841**	**(May 13)**	0	(June 11)
2013	**110**	**(May 2)**	35	(June 1)
2014	0	(April 22)	**514**	**(May 21)**
2015	**579**^a^	**(May 11)**	12	(June 10)

**Notes.**

a Data were obtained on May 10 instead of May 11 due to the effects of the typhoon.

**Figure 7:**
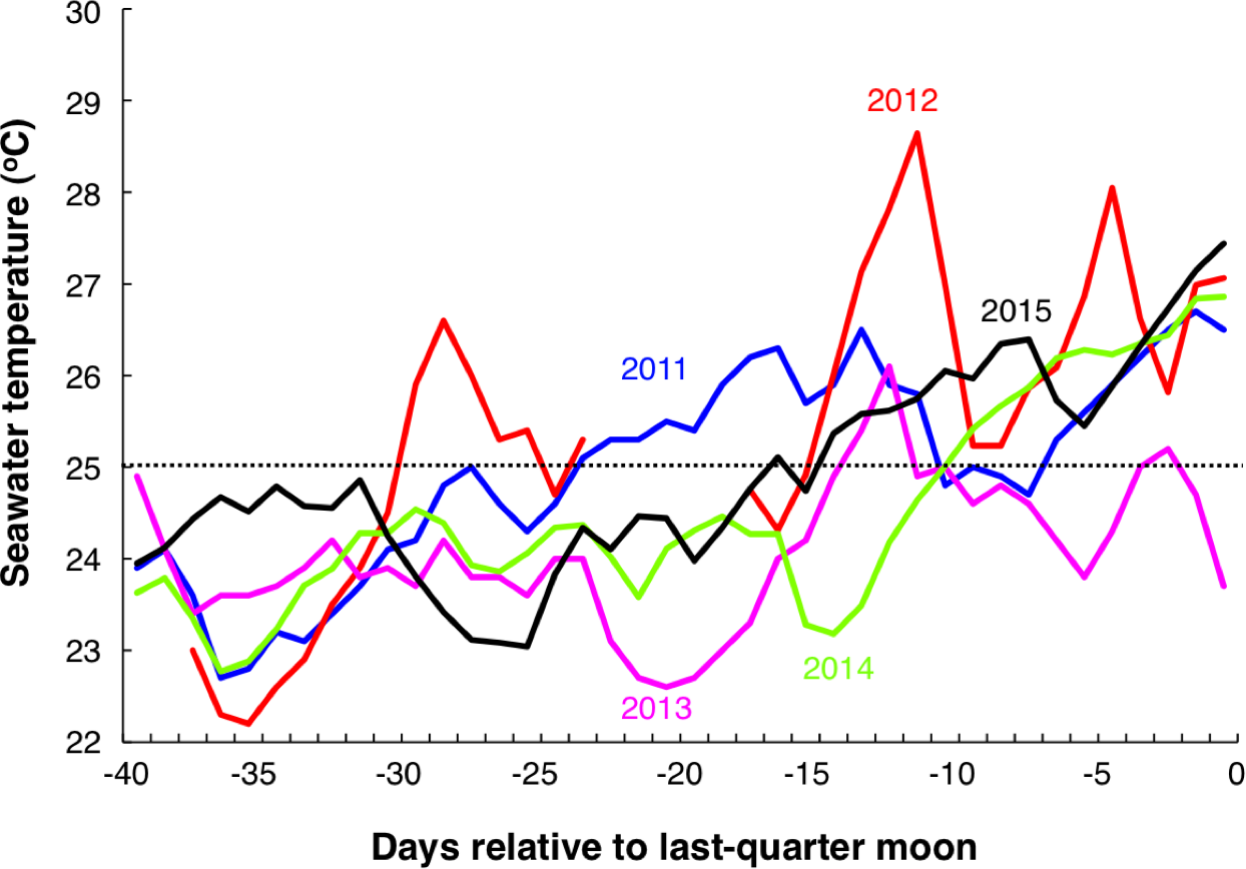
Seawater temperature profiles during 40 days before the last-quarter moon (2011 May 25, 2012 May 13, 2013 May 2, 2014 May 21 and 2015 May 11). Horizontal dotted line represents the 25 °C seawater temperature. Some profiles in 2012 (red line) were not collected due to maintenance of the data collection system. Data was provided by International Coral Reef Research and Monitoring Center.

**Figure 8:**
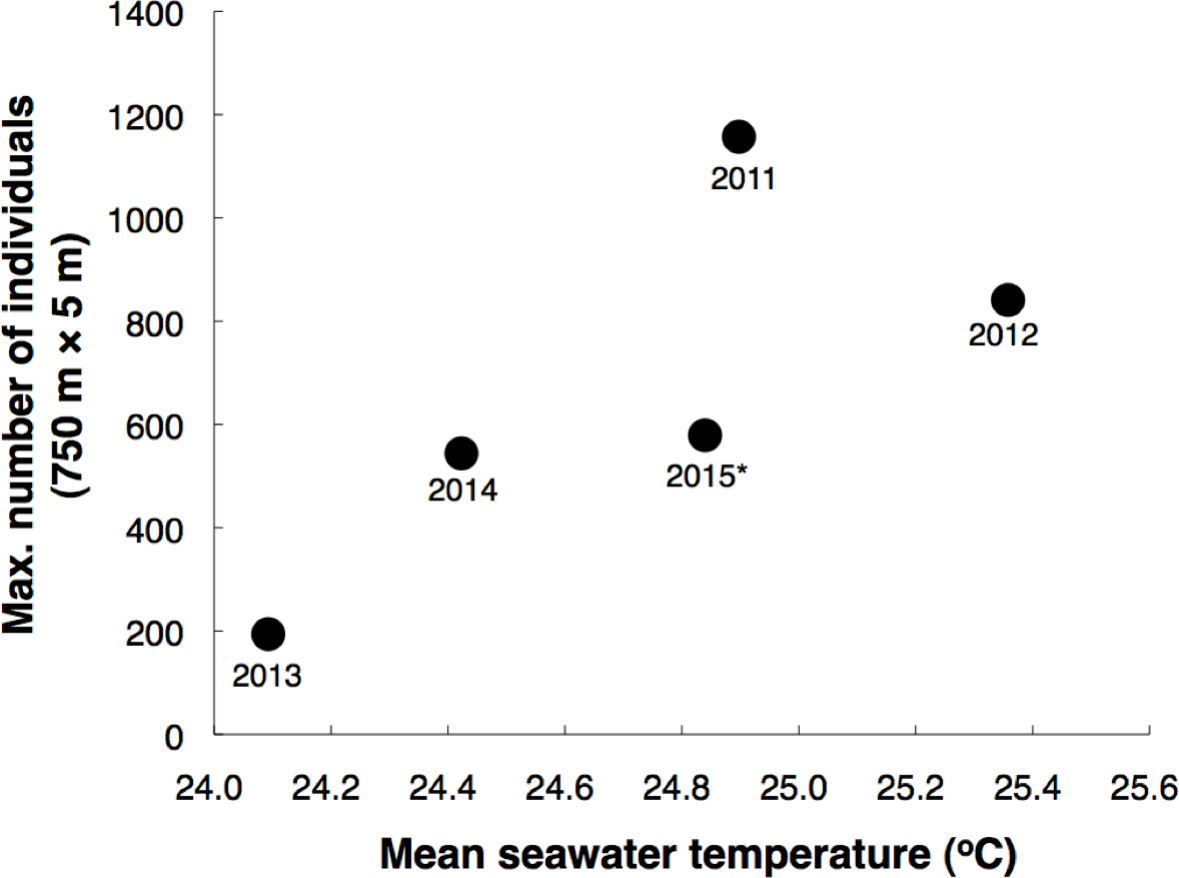
Relationship between mean seawater temperature during 40 days before the last-quarter moon and maximum number of *Epinephelus ongus* recorded in a single day at the 750 m × 5 m control transect. (Transect H: for location, see Fig. 2). *, since data could not be collected due to the typhoon which hit at the last-quarter moon in May 2015, the data that obtained 1 day before the last-quarter moon is shown.

Seawater temperature profiles revealed that the seawater temperature in 2013 dramatically dropped around 20 days and around 6 days before the last-quarter moon, and the seawater temperature was still under 25 °C at the last-quarter moon (Fig. 7). In contrast, although the seawater temperature dropped around 14 days before the last-quarter moon in 2014, the seawater temperatures were over 25 °C at the last-quarter moon in 2011, 2012, 2014 and 2015 (Fig. 7). Although it was not significant, a positive trend between mean seawater temperature (between 40 days before and the last-quarter moon) and maximum number of individuals at the 750 m × 5 m control transect was found (Fig. 8).

### Male–female ratio at the spawning ground

**Figure 9:**
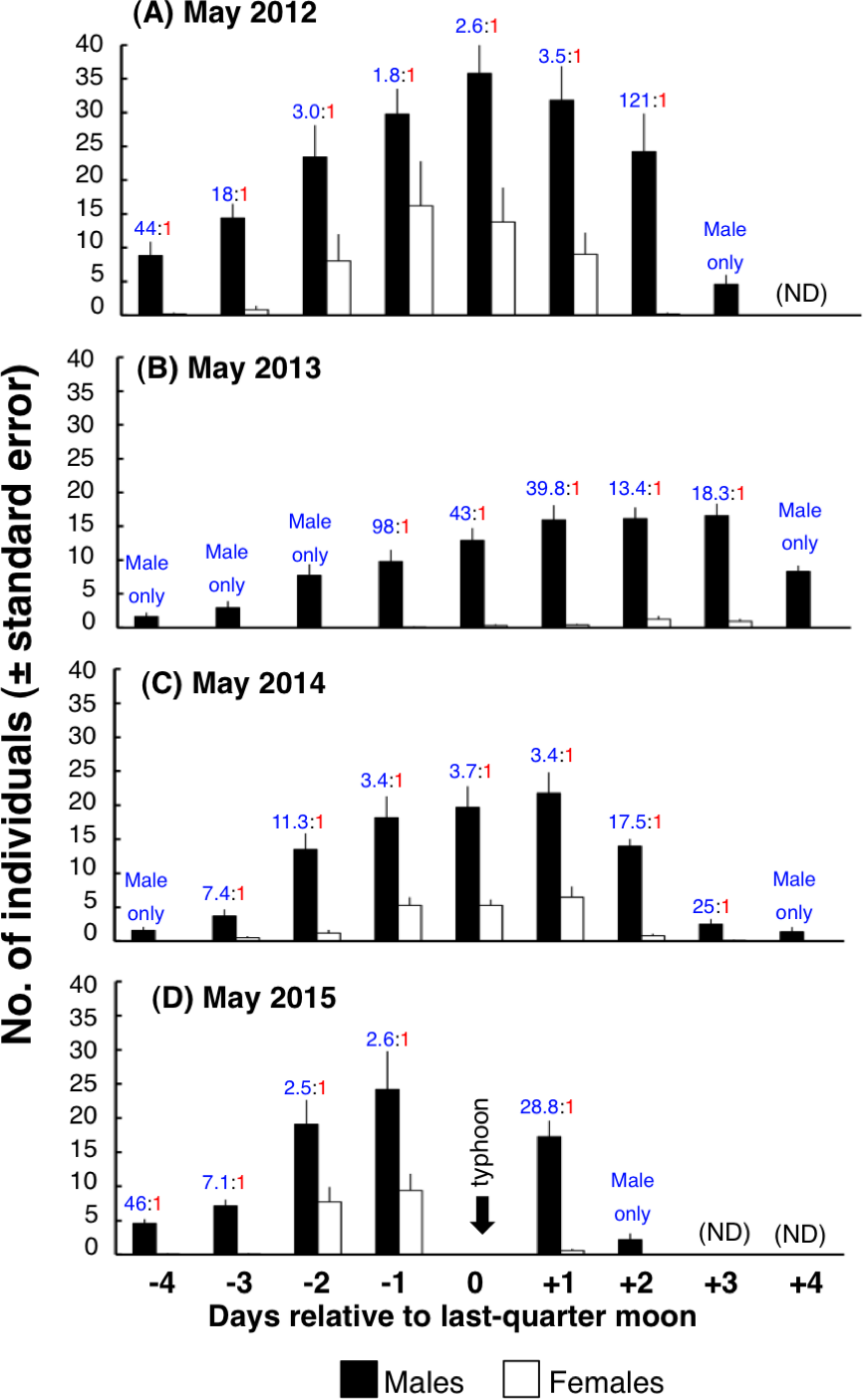
Daily changes in density of males and females on 20 m × 5 m transects. (*n* = 5 in May 2012, *n* = 10 in May 2013–May 2015). Data could not be collected due to the typhoon, which hit at the last-quarter moon in May 2015 (black arrow). Numbers on bars represent the proportion of males to females (male:female). (ND), data were not collected.

The peak of the density was observed around the last-quarter moon for both males and females (Fig. 9). In May 2012, May 2014 and May 2015, c.a. 3 males to 1 female were found around the last-quarter the moon. In May 2013, over 13 males to 1 female were found around the last-quarter moon.

### Relationship between male density and female density at the spawning ground

**Figure 10:**
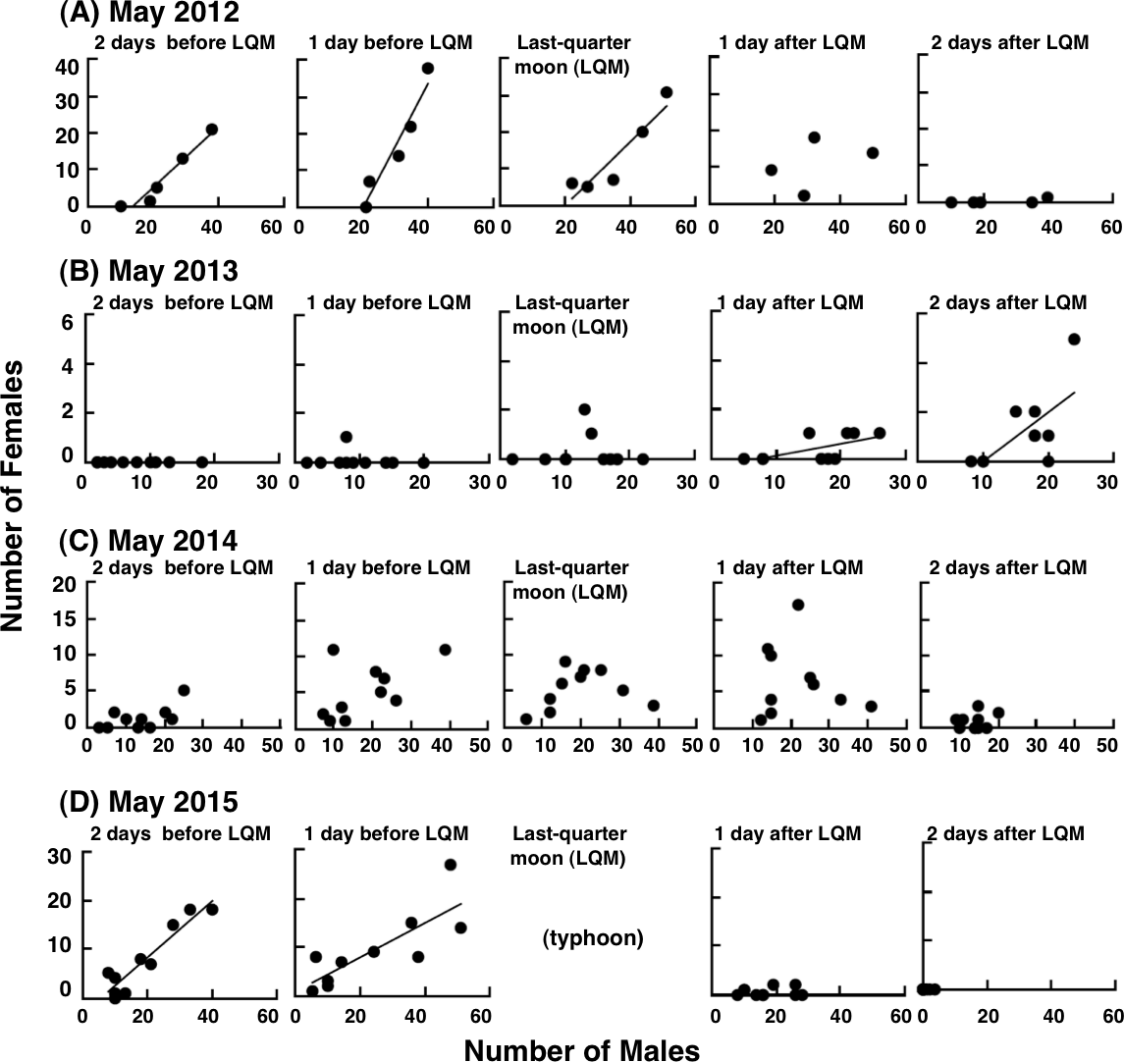
Daily change in relationship between the number of males and number of females around the last-quarter moon on 20 m × 5 m transects during the spawning periods. (*n* = 5 in May 2012, *n* = 10 in May 2013–May 2015). The results of the simple linear regression analysis are also shown for the significant positive relationships (*p* < 0.05). Data could not be collected due to the effects of the typhoon at the last-quarter moon in May 2015. LQM, last-quarter moon.

There were significant positive relationships between the male density and female density in May 2012 (two days before, one day before last-quarter moon, and the last-quarter moon phase), May 2013 (one day after and two days after the last-quarter moon) and May 2015 (two days before and one day before the last-quarter moon) (Figs. 10A, 10B, 10D). In May 2014, no significant relationship was found during the higher male/female ratio period (Fig. 10C).

## Discussion

### Spatial distribution at the spawning ground in the spawning period

The present study is the first study to clarify the spatial distribution of spawning aggregation of *Epinephelus ongus*. Some studies have shown that grouper spawning aggregations form at the convex-shaped edge of reef channels (Kobara & Heyman, 2008; Kobara & Heyman, 2010; Golbuu & Friedlander, 2011; Colin, 2012). Similar results were obtained in the present study that *E. ongus* density was markedly higher at the convex edge of the southern part of the Yonara Channel.

Colin (2012) showed that the highest density in the spawning aggregations of three grouper species (*E. polyphekadion*, *E. fuscoguttatus* and *Plectropomus areolatus*) was less than 10 individuals per 100 m^2^ in Palau. Golbuu & Friedlander (2011) found in Ngerumelkaol Channel in Palau that the density of the three grouper species was 10.28, 3.80 and 2.15 individuals per 100 m^2^ for *P. areolatus*, *E. fuscoguttatus* and *E. polyphekadion*, respectively. Samoilys & Squire (1994) showed the highest density of *Plectropomus leopardus* was 4.4 per 100 m^2^ on the northern Great Barrier Reef. In contrast, Rhodes & Sadovy (2002) showed that over 500 individuals per 100 m^2^ were found for *E. polyphekadion* in Pohnpei, Micronesia. Thus, it is suggested that the maximum density of spawning aggregations for epinephelid species is species-specific and location-specific, although another possible explanation is the different degree of exploitation of the spawning aggregations among the study sites.

The present study also revealed that the relative current velocity as well as coverage of rocks had significant positive effects on the density of the spawning aggregation of *E. ongus*. Although *E. ongus* inhabits areas dominated by branching *Acropora* in the non-spawning period outside the spawning ground (Nanami et al., 2013b), the greater density of *E. ongus* inside the spawning ground was found in the area with a greater current velocity with rocks. In contrast, lower density or almost no individuals were found at the northern parts of the spawning ground even though these parts were mainly covered by branching *Acropora*. It is suggested that the positive effect of the greater current velocity on the *E. ongus* density is due to the effective transportation of their zygotes. The greater current velocity might also contribute to reduce the extent of their released eggs from being preyed on by other species. In contrast, for spawning behavior, *E. ongus* was observed to spawn during the slack tide (Nanami et al., 2013a). These results suggest that *E. ongus* select aggregation sites with a greater current velocity to transport egg to the outside of the spawning ground as well as to avoid egg predation, however for actual mating *E. ongus* select the spawning time to enhance the mixing and fertilization of the eggs and sperm before offshore transport of eggs.

### Inter-annual variation in degree of aggregation

Some previous studies have shown inter-annual variation in aggregation size (Nemeth, 2005; Golbuu & Friedlander, 2011; Rhodes et al., 2014; Gouezo et al., 2015). The maturation of the groupers, especially for female gonad development, is affected by seawater temperature (Teruya et al., 2008; Ohta & Ebisawa, 2009). These previous studies suggested that the warm seawater temperature before the spawning season promotes gonad development of groupers. The results of the present study suggested that gonads may not have been sufficiently developed in 2013 due to the low seawater temperature and this led to the relatively small number of arrivals into the spawning ground in that year. Such annual variations in seawater temperature might be caused by annual variation of climate and weather conditions. Long-term monitoring is needed to clarify the relationship between seawater temperature and degree of aggregation.

### Male–female ratio and relationship between male density and female density

The number of females was less than males for *E. ongus* (male:female ranged from >13:1 to c.a. 3:1). Under this condition, females would be able to select better male individuals for reproduction (e.g., large male with a greater amount of sperm), since *E. ongus* is pair-spawner (Nanami et al., 2013a). In contrast, some previous studies have shown that the sex ratios at aggregation sites were female-biased. The male–female ratios (male:female) previously observed were 1:5–1:3 for *E. striatus* (Colin, 1992), 1:5.6–1:7.1 for *E. guttatus* (Shapiro, Sadovy & McGehee, 1993) and 0.2:1–0.3:1 for *E. polyphekadion* (Rhodes & Sadovy, 2002).

During the 4 years of survey, a significant positive relationship between the male density and female density at the spawning ground in the spawning period was observed for three years (May 2012, May 2013 and May 2015). This positive relationship might be due to the fine-scale (within tens of meters) site selection by females. Since females arrived later than males, females could select sites with a high male density. As the purpose of spawning aggregations is reproduction (Molloy, Côte & Reynolds, 2012), the fine-scale site selection by females related to male density would potentially increase the reproductive success. Another possible reason is that both males and females might simply select sites with greater current velocity and greater coverage of rocks. However, if both male and female simply select the site with better environmental characteristics, it is likely that males and females would arrive at the spawning ground at the same timing. Although the results of the present study is not conclusive about this relationship, the present study does provide a useful insight as to the reason why there is a sexual difference in timing of arrival for *E. ongus*. However, the relationship between male density and female density was not found in May 2014, indicating that the relationship might annually be variable. Further study is needed to clarify the relationship between male density and female density in relation to sexual difference in arrival timing at the spawning ground.

### Implications for effective management

From the results of the present study, effective management of *E. ongus* in terms of spawning ground protection should be considered as follows. The critical site such as edge of the reef channel should be included within the protected area, and it was confirmed that this has been currently achieved in the Yonara Channel. Although the protection duration is not necessarily all-year round, the spawning period, especially May, should be included in the present study site. Since males arrive earlier and stay longer within the spawning ground (Nanami et al., 2014), the protection duration should be also considered based on the behavior of the males (protection duratio *n* = 20 days at present). Males would be more vulnerable and the female/male ratio would increase by temporal sex-specific fishing pressure without spawning ground protection (Sadovy de Mitcheson & Erisman, 2012). Since *E. ongus* is a protogynous hermaphrodite (Ohta & Ebisawa, 2016), larger-sized males would be affected by a higher fishing pressure without spawning ground protection. Consequently, sperm limitation among the *E. ongus* population might occur under high fishing pressure, since *E. ongus* is a pair-spawner (Nanami et al., 2013a). Long-term monitoring of the male–female ratio should be conducted to estimate the status of the spawning aggregation for *E. ongus*. In addition, expansion of the protected area to include the migration routes would be optimal for the protection of the spawning aggregation of *E. ongus*. To do this, precise data about migration routes need to be clarified in the future.

##  Supplemental Information

10.7717/peerj.3000/supp-1 Figure S1 Result of principal component analysis (PCA) Results showing the relationship between the relative current velocity and the PCA scores (A), between the substrate characteristics and the PCA scores (B) and between the depth and the PCA scores (C) on PC axis 1 and PC axis 3.

10.7717/peerj.3000/supp-2 Video S1 Video S1

10.7717/peerj.3000/supp-3 Video S2 Video S2

10.7717/peerj.3000/supp-4 Supplemental Information 4 Raw data for Figs. 4 and 5, Fig. S1 and Tables 1 and 2

10.7717/peerj.3000/supp-5 Supplemental Information 5 Fig. 6 raw data

10.7717/peerj.3000/supp-6 Supplemental Information 6 Fig. 8 raw data

10.7717/peerj.3000/supp-7 Supplemental Information 7 Fig. 9 raw data

10.7717/peerj.3000/supp-8 Supplemental Information 8 Fig. 10 raw data

## Additional Information and Declarations

### Data Availability

The following information was supplied regarding data availability: The raw data has been supplied as a Supplemental File.
