# Supplementary figures and images for: Spawning aggregation of white-streaked grouper Epinephelus ongus: spatial distribution and annual variation in the fish density within a spawning ground
Source: PeerJ. 2017 Feb 14;5:e3000. doi: 10.7717/peerj.3000 (PMC5312569; doi:10.7717/peerj.3000)

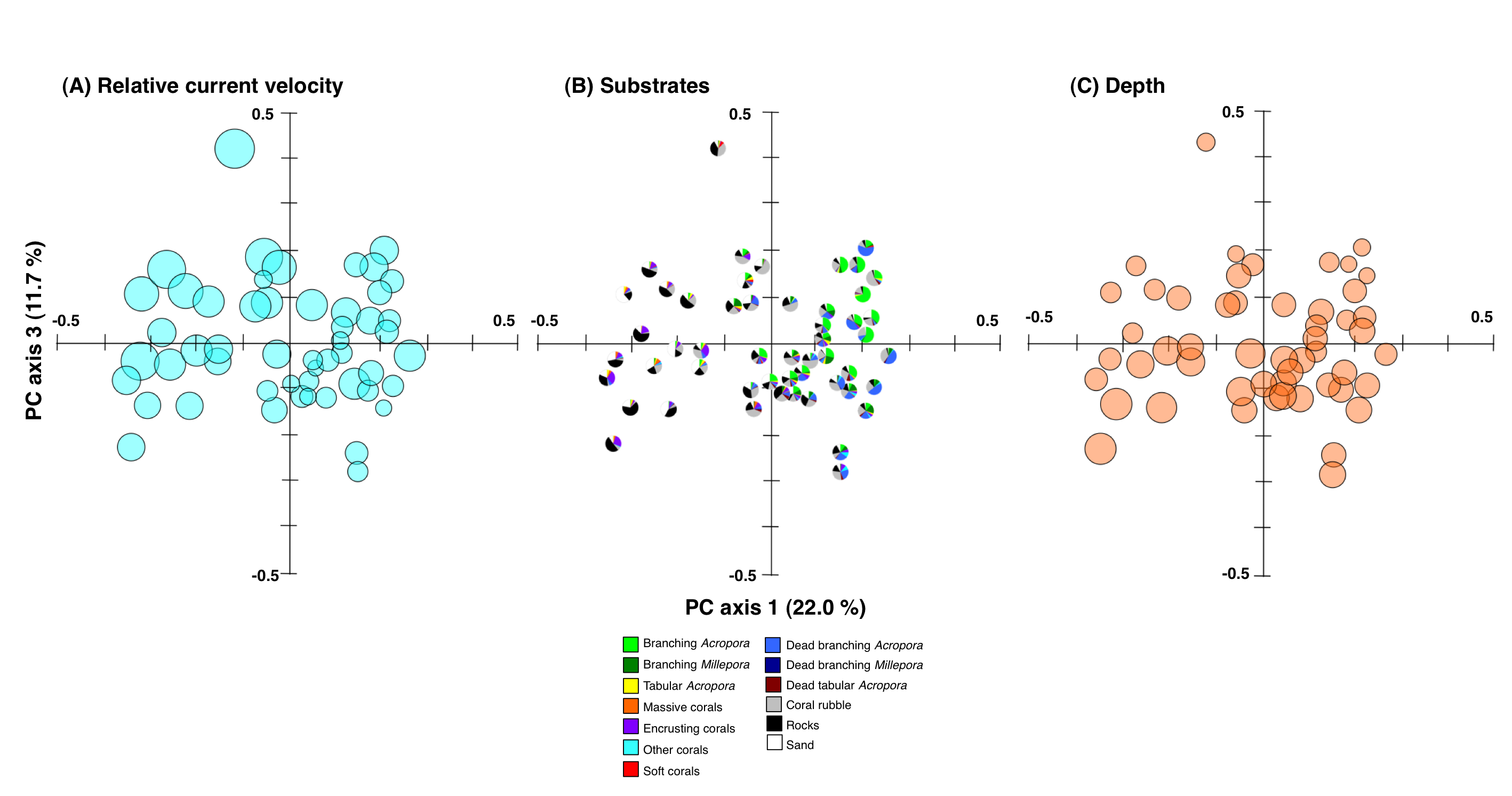

Supplement: Figure S1 — Results showing the relationship between the relative current velocity and the PCA scores (A), between the substrate characteristics and the PCA scores (B) and between the depth and the PCA scores (C) on PC axis 1 and PC axis 3. [file peerj-05-3000-s001.png]
